# Case report: Common clonal origin of concurrent langerhans cell histiocytosis and acute myeloid leukemia

**DOI:** 10.3389/fonc.2022.974307

**Published:** 2022-09-16

**Authors:** Shintaro Kazama, Kazuaki Yokoyama, Toshimitsu Ueki, Hiroko Kazumoto, Hidetoshi Satomi, Masahiko Sumi, Ichiro Ito, Nozomi Yusa, Rika Kasajima, Eigo Shimizu, Rui Yamaguchi, Seiya Imoto, Satoru Miyano, Yukihisa Tanaka, Tamami Denda, Yasunori Ota, Arinobu Tojo, Hikaru Kobayashi

**Affiliations:** ^1^ Department of Hematology, Nagano Red Cross Hospital, Nagano, Japan; ^2^ Division of Molecular Therapy, Institute of Medical Science, Advanced Clinical Research Center, The University of Tokyo, Tokyo, Japan; ^3^ Department of Diagnostic Pathology and Cytology, Osaka International Cancer Institute, Osaka, Japan; ^4^ Department of Pathology, Nagano Red Cross Hospital, Nagano, Japan; ^5^ Department of Applied Genomics, Research Hospital, Institute of Medical Science, University of Tokyo, Tokyo, Japan; ^6^ Molecular Pathology and Genetics Division, Kanagawa Cancer Center Research Institute, Yokohama, Japan; ^7^ Division of Health Medical Data Science, Health Intelligence Center, Institute of Medical Science, University of Tokyo, Tokyo, Japan; ^8^ Division of Cancer Systems Biology, Aichi Cancer Center Research Institute, Nagoya, Japan; ^9^ Department of Integrated Data Science, Medical and Dental Data Science Center, Tokyo Medical and Dental University, Tokyo, Japan; ^10^ Department of Diagnostic Pathology, IMSUT Hospital, Institute of Medical Science, The University of Tokyo, Tokyo, Japan; ^11^ Department of Data Science and Faculty Affairs, Tokyo Medical and Dental University, Tokyo, Japan

**Keywords:** langerhans cell histiocytosis, acute myeloid leukemia, NRAS, MAPK pathway, histiocytic disorders, dendritic cells, BRAF V600E, inflammatory myeloid neoplasm

## Abstract

Langerhans cell histiocytosis (LCH) and acute myeloid leukemia (AML) are distinct entities of blood neoplasms, and the exact developmental origin of both neoplasms are considered be heterogenous among patients. However, reports of concurrent LCH and AML are rare. Herein we report a novel case of concurrent LCH and AML which shared same the driver mutations, strongly suggesting a common clonal origin.An 84-year-old female presented with cervical lymphadenopathy and pruritic skin rash on the face and scalp. Laboratory tests revealed pancytopenia with 13% of blasts, elevated LDH and liver enzymes, in addition to generalised lymphadenopathy and splenomegaly by computed tomography. Bone marrow specimens showed massive infiltration of MPO-positive myeloblasts, whereas S-100 and CD1a positive atypical dendritic cell-like cells accounted for 10% of the atypical cells on bone marrow pathology, suggesting a mixture of LCH and AML. A biopsy specimen from a cervical lymph node and the skin demonstrated the accumulation of atypical cells which were positive for S-100 and CD1a. LCH was found in lymph nodes, skin and bone marrow; AML was found in peripheral blood and bone marrow (AML was predominant compared with LCH in the bone marrow).

Next generation sequencing revealed four somatic driver mutations (*NRAS*-G13D, *IDH2*-R140Q, and *DNMT3A*-F640fs/-I715fs), equally shared by both the lymph node and bone marrow, suggesting a common clonal origin for the concurrent LCH and AML. Prednisolone and vinblastine were initially given with partial response in LCH; peripheral blood blasts also disappeared for 3 months. Salvage chemotherapy with low dose cytarabine and aclarubicin were given for relapse, with partial response in both LCH and AML. She died from pneumonia and septicemia on day 384. Our case demonstrates a common cell of origin for LCH and AML with a common genetic mutation, providing evidence to support the proposal to classify histiocytosis, including LCH, as a myeloid/myeloproliferative malignancy.

## Introduction

Langerhans cell histiocytosis (LCH) and acute myeloid leukemia (AML) are distinct entities of blood neoplasms, and the exact developmental origin of both neoplasms is considered to be heterogenous among patients (1–6). However, to our knowledge, only ten cases of concurrent LCH and AML have been reported so far (2, 3, 7–11). Herein we report a novel case of concurrent AML and LCH which shared the same driver mutations, strongly suggesting a common clonal origin.

## Case description

**Figure 1:**
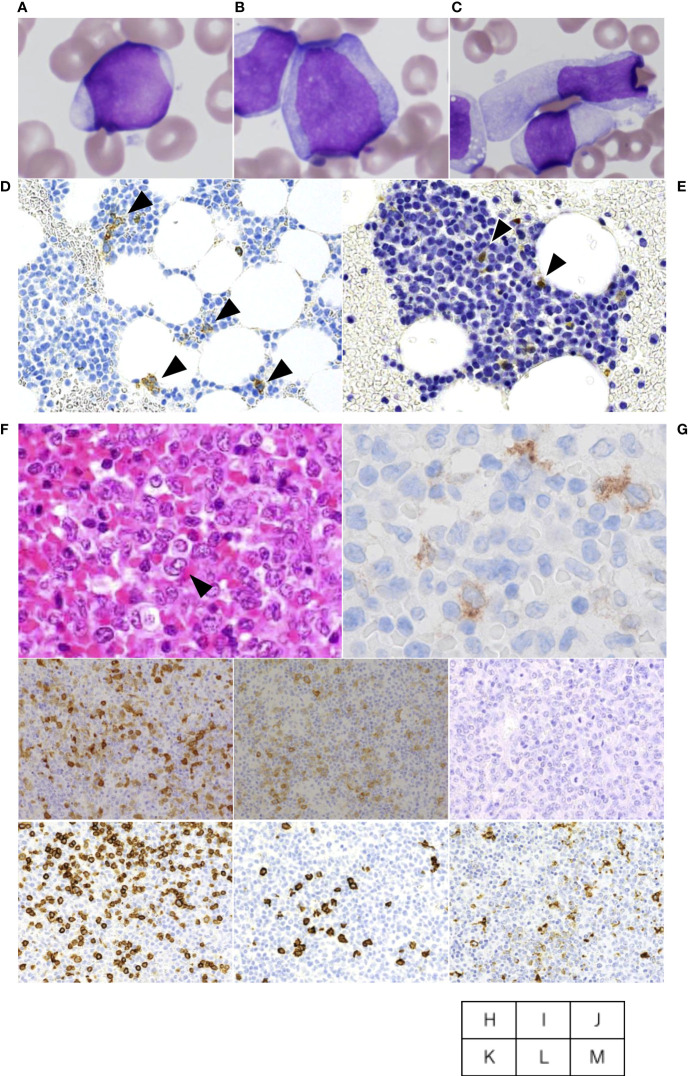
Cervical lymph node biopsy and bone marrow aspiration. The aspiration smears of bone marrow show myeloblastic cells accounted for 53.5% **(A)** and monoblastic cells accounted for 13.8% **(B)**, whereas dendritic cell-like atypical cells are also found at a frequency of 9.1% **(C)**. On bone marrow pathology, CD1a **(D)**- and S-100 **(E)**-positive atypical cells accounted for 10% of the blasts. Cervical lymph node biopsy specimen shows atypical cells with indented or folded nuclei like one indicated by arrowhead or other ones with mild folding **(F)**, and immunostaining was Langerin-partially positive **(G)**, S-100-positive **(H)**, CD1a-positive **(I)**, MPO-negative **(J)**. Around the atypical cells were scattered mononuclear cells positive for CD3 **(K)**, CD20 **(L)**, and CD68 **(M)** in the periphery.

An 84 year-old female presented with cervical lymphadenopathy and pruritic skin rash on the face and scalp. She had no past history or family history of hematological or solid organ malignancies or other treatments. She had no fever but multiple lymph nodes were palpated bilaterally in the neck, axilla, and inguinal region. Laboratory tests revealed pancytopenia (WBC 2,520/μL including 13% blasts, 32% neutrophils, 6% eosinophils, 0% basophils, 3% monocytes, 46% lymphocytes, Hemoglobin (Hb) of 10.9 g/dL, and a platelet count of 92,000/μL) and elevated LDH (604 U/L) and liver enzymes (AST 51 U/L, ALT 39 U/L). Systemic lymphadenopathy and splenomegaly, but no other abnormal findings, were noted on computed tomography. Bone marrow specimens showing a massive infiltration of MPO-positive blasts including 53.5% of myeloblastic cells and 13.8% of monoblastic cells indicated the diagnosis of AML. Whereas, 9.1% of cellular components were occupied by MPO-negative atypical dendritic cell-like cells (**Figures 1A–C**). On flow cytometric analysis, monoclonal cells were positive for CD13, CD33, CD34, CD38, CD7, and TdT. On bone marrow pathology, S-100 and CD1a-positive atypical cells accounted for 10% of the blasts (**Figures 1D, E**), suggesting a mixture of AML and LCH. A biopsy specimen from a cervical lymph node demonstrated the accumulation of atypical round or horseshoe-shaped cells with indented or folded nuclei which were positive for S-100 and CD1a, and negative for MPO and CD34, confirming the diagnosis of LCH (**Figures 1F, H–J**). AML cells were absent not only on pathology, but also on flow cytometry in lymph nodes. Atypical cells were partially positive for langerin (**Figure 1G**), not suggestive of indeterminate cell histiocytosis (ICH). Around the atypical cells were scattered mononuclear cells positive for CD3, CD20, and CD68 (**Figures 1K–M**). Simultaneously, skin biopsy demonstrated atypical cells with an immunohistochemical profile similar to that in the lymph nodes, suggesting skin invasion of LCH. The atypical cells were negative for MPO and CD34, suggesting absence of skin invasion by the AML. The G-banding assay revealed a normal karyotype in both the bone marrow and lymph nodes.

LCH was found in lymph nodes, skin and bone marrow; AML was found in peripheral blood and bone marrow (AML was predominant compared with LCH in the bone marrow). Elevated liver enzymes and splenomegaly suggested the presence of hepatic and splenic lesions, but which tumor was responsible could not be determined.

## Diagnostic assessment

Taken together, the patient was diagnosed with concurrent LCH and AML. Next generation sequencing with the TruSight Myeloid Sequencing Panel (Illumina) revealed four somatic driver mutations (*NRAS*-G13D, *IDH2*-R140Q, and *DNMT3A*-F640fs/-I715fs), equally found in both the lymph node and bone marrow (**Table 1**). *BRAF*-V600E was negative, which was also confirmed by immunohistochemistry staining (data not shown). The mutant allele frequencies were 24.6%-33.0% and 27.0%-38.5% in the lymph node and bone marrow, respectively. Assuming each mutant allele to be a single hit, 50-70% of tumor cells harbored each mutation, suggesting a common clonal origin of concurrent LCH and AML.

**Table 1:** Results of genomic analysis of cervical lymph node biopsy and bone marrow aspiration by panel analysis.

**Bone marrow** (Total somatic mutations 3,885 ⊳ Driver mutations 4)				
Amino acid mutations	*NRAS* p.G13D	*IDH2* p.G13D	*DNMT3A* p.F640fs	*DNMT3A* p.I715fs
			
Mutant allele frequency (%)	32.8	24.6	33.0	28.7
**Lymph nodes** (Total somatic mutations 2,468 ⊳ Driver mutations 4)				
Amino acid mutations	*NRAS* p.G13D	*IDH2* p.R140Q	*DNMT3A* p.F640fs	*DNMT3A* p.I715fs
		
Mutant allele frequency (%)	30.9	27.0	37.1	38.5

**Figure 2:**
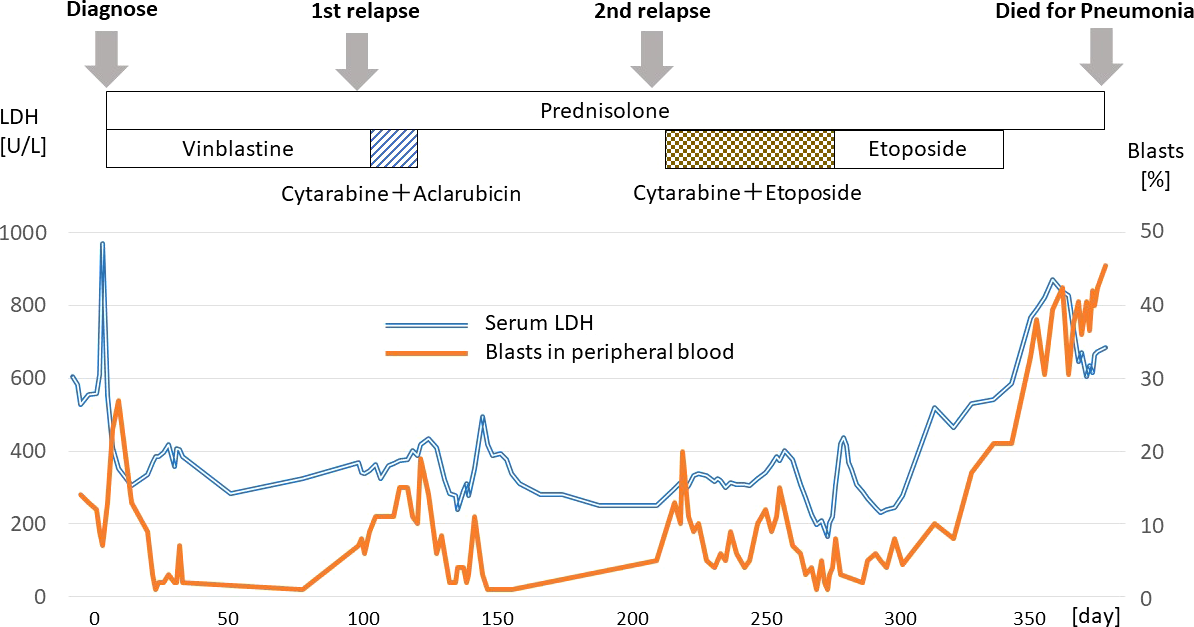
The timeline of diagnosis and treatment.

Prednisolone and vinblastine were initially given with partial response in LCH, additionally peripheral blood blasts disappeared for 3 months. From day 120, low dose cytarabine and aclarubicin were administered as a salvage chemotherapy with partial response in both LCH and AML. Subsequently, from day 253, the patient received low dose cytarabine and etoposide, which did not give rise to a durable response, and she died from pneumonia and septicemia on day 384 (**Figure 2**).

## Discussion

Histiocytic disorders are a group of rare diseases characterized by organ infiltration by macrophages, dendritic cells, and monocytes (12). Gene mutations have been identified in a number of histiocytoses, including LCH and non-LCH (e.g., Erdheim-Chester disease (ECD), indeterminate cell histiocytosis, and histiocytic sarcoma) (5, 13–20), most of which are mutations in genes encoding proteins in the mitogen-activated protein kinase (MAPK) pathway (21). Therefore, the pathogenesis of histiocytic disorders, including LCH are mainly attributed to unregulated activation of the MAPK pathway (1, 22), and these disorders are clonal neoplastic diseases caused by this (23, 24). Activating mutations of MAPK pathway members are almost mutually exclusive. Among these, *BRAF* is a major target of mutation, and *BRAF*-V600E is most commonly observed in LCH (1). In contrast, mutation of *NRAS*, another member of the MAPK pathway, is rare in LCH, but one report stated that *NRAS* mutations were present in 40% of pulmonary LCH (25). Furthermore, genetic mutations in the MAPK pathway members including *NRAS* were identified in 57% of Langerhans cell sarcomas (26). *NRAS* mutations were more common in AML (10%) than LCH, and, in combination with *IDH2*, *DNMT3A* and other mutations, contribute to the pathogenesis of AML (27–29).

Cases of LCH combined with malignant neoplasms are rare and generally the subject of isolated case reports (2, 3). Most reports of AML associated with LCH are treatment-related AML after treatment for previous LCH. Only ten cases of simultaneous diagnosis of LCH and AML have been reported to date, most of which were characterized by generalized LCH, monocytic leukemia as the predominant type of associated AML, and poor prognosis, as in the present case (2, 3, 7–11). On the other hand, a high concomitant rate of myeloid neoplasm has been reported in non-LCH (e.g., 10.1% in ECD) (30, 31).

The exact cell of origin of histiocytic disorders, including LCH, is unknown. Allelic assessment of Langerhans cells in LCH clearly distinguished them from skin Langerhans cells (4), as *BRAF*-V600E mutations were identified in a subset of dendritic cells, mature monocytes, myeloid progenitor cells, and CD34+ cells in LCH and ECD patients. Therefore, it can be estimated myeloid progenitor cells are the cell of origin for histiocytic disorders (5, 32, 33). However, clinical and experimental evidence is lacking.

Recently, advances in genetic analysis have led to the discovery of commonly mutated genes in cases of histiocytic disorders and myeloid neoplasms, and the existence of a common cellular origin of histiocytic disorders and myeloid neoplasms has been proposed. In a review by Kemps PG et al. (34), mutated genes in histiocytic disorders and myeloid neoplasms have been reported in 30 cases (29, 33–55). A total of 31 cases, including one additional case of a common mutation found in LCH and primary myelofibrosis (39), including the present case are shown in **Table 2**. There have been five cases, of LCH occurring concurrently with a myeloid neoplasm, including the present case. Gene mutations of the MAPK pathway were frequently detected in LCH and non-LCH (30, 34, 35, 37, 40–44). There have been 5 cases of shared *NRAS* mutations, and one of them was at the same locus as the present case (*NRAS* p.G13D) (42). In this case, an adult female was diagnosed with ECD from skin lesions, and bone marrow biopsy revealed chronic myelomonocytic leukemia. The only mutation detected was *NRAS* p.G13D, which was common to both lesions, but *BRAF*-V600E was not examined.

**Table 2:** Overview of reported cases with histiocytic disorders and additional myeloid neoplasms bearing the same genetic alteration(s).

No.	pediatric/adult	Histiocytic neoplasms	associated myeloid neoplasms	shared driver mutations	Reference
1	A	LCH	CMML	*BRAF*p.V600E	(34)
2	A	LCH	AML NOS	*ASXL1,IDH2 and STAG2* mutations	(35)
3	A	LCH	AML NOS	Trisomy 8,*KRAS*pA146T	(36)
4	A	LCH	PMF	*JAK2*p.V617F	(37)
5	A	LCH	PMF	*JAK2*p.V617F	(38)
6	A	Mixed LCH/ECD	ET	*JAK2*p.V617F	(29)
7	A	Mixed LCH/ECD	AML-M4	*TET2*p.L1819X and *SRSF2*p.L95P	(39)
8	A	ECD	AML-M5	*NRAS*p.Q61R	(33)
9	A	ECD	AML-M5	*BRAF*p.V600E	(33)
10	A	ECD	AML NOS	*BRAF*p.V600E	(40)
11	A	ECD	CMML	*BRAF*p.V600E, *TET2* and *SRSF2* mutations	(40)
12	A	ECD	CMML	*KRAS*p.G12D and *ASXL1*p.G642fs	(41)
13	A	ECD	CMML	*KRAS*p.G12D and *DNMT3A*p.Y623fs	(41)
14	A	ECD	CMML	*KRAS*p.G12D.*ASXL1*p.Y591X	(42)
15	A	ECD	CMML	*NRAS*p.G13D	(41)
16	A	ECD	CMML	*NRAS*p.Q61R	(29)
17	A	ECD	CMML	*NRAS*p.Q61R	(40)
18	A	ICH	CMML	*NRAS*p.G12V	(33)
19	A	ICH	CMML	*KRAS*p.G12R	(43)
20	A	ICH	CMML	*TET2*p.Q1466X and p.Q1523X,*ASXL1*p.K618X and *ZRS2*p.Q100X	(44)
21	P	JXG	JMML	*PTPN1*p.E76K	(45)
22	A	HS	CMML	*KRAS*p.A59E	(33)
23	N/A	HS	CMML	*TP53* mutation	(46)
24	A	HS	MDS	*TP53* and BCOR mutations	(47)
25	A	Atypical non LCH	AML MO	*RUNX1*p.R166X and p.P425L	(48)
26	A	MPDCN	MDS-MLD	*PTPN11*p.R501K	(49)
27	A	BPDCN	AML NOS	*TET2*p.C1642fs and p.A1810fs and *SRSF2*p.P95H	(50)
28	A	BPDCN	CMML	*TET2*p.G523fs, *SRSF2*p.P85L,*PHF6*p.Q251H	(51)
29	A	BPDCN	CMML	*TET2* p.Y1244fs and p.Q1810X and *SRSF2*p.P95H	(52)
30	N/A	BPDCN	CMML	*TET2* mutation	(53)
31	A	BPDCN	MDS-RARS	*TET2* mutation	(54)
32	A	LCH	AML	*NRAS*p.G13D,*IDH2*p.R140Q,*DNMTA*p.F640fs and p.1715fs	The present case

P, paediatric; A, adult; N/A, not available; LCH, Langerhans cell histiocytosis; ECD, Erdheim Chester disease; ICH, indeterminate cell histiocytosis; JXG, Juvenile xanthogranuloma; HS, histiocytic sarcoma; non-LCH, non-Langerhans cell histiocytosis; MPDCN, mature plasmacytoid dendritic cell neoplasm; BPDCN, blastic plasmacytoid dendritic cell neoplasm; CMML, chronic myelomonocytic leukemia; AML, acute myeloid leukemia; NOS, not otherwise specified; PMF, primary myelofibrosis; ET, essential thrombocytosis; JMML, juvenile myelomonocytic leukaemia; MDS, myelodysplastic syndromes; MLD, multilineage dysplasia; RARS, refractory anaemia with ring sideroblasts.

Five cases of myeloid neoplasms have been suggested to have a common origin with LCH (2 cases of AML, 2 cases of polycrythemia vera, and 1 case of chronic myelomonocytic leukemia), thus, this is the third report of common gene mutation between LCH and AML. In the other two reports of shared mutation between LCH and AML, both adult males were diagnosed with LCH on skin biopsy and AML from bone marrow biopsy. One patient shared *ASXL1*, *IDH2*, and *STAG2* mutations (36), and the other shared *KRAS* mutation and trisomy 8 (37). Of note, in both cases, *BRAF*-V600E mutation was detected only in LCH cells. In the present case, allele frequencies of 4 driver mutations were comparable to each other as well as between LCH (lymph node) and AML (bone marrow), no genetic mutations present in only AML or LCH were detected, making it difficult to dissect the developmental process of LCH and AML. Thus, the present case provides important evidence that myeloid progenitors are the common origin of the two neoplasms, and reaffirms the importance of MAPK pathway activation in the pathogenesis of histiocytic disorders.

On the other hand, a different biological mechanism by which leukemic progenitor cells are misdirected into LCH by environmental factors was considered. This disease is an inflammatory myeloid neoplasm with features of both abnormal reaction processes and neoplastic processes. De Graaf et al. reported that inflammatory cytokines are expressed in LCH lesions (56). Kannourakis et al. extracted and cultured monocytes from the eosinophilic granuloma tissue of LCH patients, and found that such monocytes highly express inflammatory cytokines (57). In addition to a cytokine storm in local lesions, the levels of several pro-inflammatory cytokines in the serum of LCH patients are high, suggesting that cytokines are associated with the pathogenesis of LCH (58). LCH cells in the target tissues of LCH patients are surrounded by lymphocytic infiltrates and multinucleated giant cells, including T cells, macrophages, eosinophils, and B cells. Cytokines derived from LCH cells and T cells are considered to regulate the differentiation, maturation, and migration of myeloid dendritic cell precursors originating from hematopoietic stem cells (59, 60). In our case, the lymph node lesions also exhibited CD3-, CD20-, and CD68-positive mononuclear cell infiltrates around the LCH cells, suggesting that the microenvironment of these inflammatory cells played a role in the induction of LCH. Additionally, LCH lesions in this case were widely distributed in lymph nodes, but organs susceptible to LCH such as bone remained intact. Considering the common AML genotype and atypical LCH phenotype, there is one possible explanation for this case, that leukemic progenitor cells which migrated into lymph nodes could have been misguided toward LCH by environmental factors including the lymph node microenvironment and soluble factors.

According to the 2016 WHO classification, histiocytic disorders, including LCH and ECD, are classified as lymphoid neoplasms (61). This classification is based on several case reports describing individual patients with secondary malignant histiocytosis clonally associated with lymphoid neoplasms (62–66). However, with the recent development of genetic analysis techniques, the number of shared mutations in histiocytosis and myeloid neoplasms has surpassed those in lymphoid neoplasms (33).

## Conclusion

In summary, our case demonstrates a common cell of origin for LCH and AML with a common genetic mutation, providing evidence to support the proposal to classify histiocytosis, including LCH, as a myeloid/myeloproliferative malignancy.

## Data availability statement

The detailed clinical data and datasets presented in this article are not publicly available due to ethical and privacy restrictions. Requests to access the datasets should be directed to the corresponding authors.

## Ethics statement

Written informed consent was obtained from the [individual(s) AND minor(s)’ legal guardian/next of kin] for the publication of any potentially identifiable images or data included in this article.

## Author contributions

SK, TU, HKa, HS, MS, II, YT, TD, YO, and HKo were involved in the description of clinical information. KY, NY, ES, RY, SI, SM, and AT were involved in the data analysis. All authors contributed to the article and approved the submitted version.

## Acknowledgments

We would like to thank Editage (www.editage.com) for English language editing.

## Conflict of interest

The authors declare that the research was conducted in the absence of any commercial or financial relationships that could be construed as a potential conflict of interest.

## Publisher’s note

All claims expressed in this article are solely those of the authors and do not necessarily represent those of their affiliated organizations, or those of the publisher, the editors and the reviewers. Any product that may be evaluated in this article, or claim that may be made by its manufacturer, is not guaranteed or endorsed by the publisher.
